# Exploring Alternatives to Polyacrylamide: A Comparative Study of Novel Polymers in the Flocculation and Dewatering of Iron Ore Tailings

**DOI:** 10.3390/polym15143019

**Published:** 2023-07-12

**Authors:** Gustavo P. Zago, Reinaldo Giudici, João B. P. Soares

**Affiliations:** 1 Chemical Engineering Department, Polytechnic School, University of Sao Paulo, Sao Paulo 05508-220, SP, Brazil; rgiudici@usp.br; 2 Chemical and Materials Engineering Department, University of Alberta, Edmonton, AB T6G 2R3, Canada; jsoares@ualberta.ca

**Keywords:** polyacrylamide, flocculation, dewatering, iron ore tailings

## Abstract

Despite being widely used in tailings treatment, polyacrylamide continues to face performance challenges. In this study, two commercial polyacrylamides with different molecular weights were used to flocculate iron ore tailings and their performance was compared with two polymers designed to treat oil sand tailings: poly(vinylbenzyl)trimethylammonium chloride and partially hydrolyzed poly(methyl acrylate) grafted onto ethylene-propylene-diene copolymer backbones. The polyacrylamide with the highest molecular weight performed better than the one with the lowest molecular weight, but its efficiency was still considerably lower than what would be desired for good solid–liquid separation. The new polymer flocculants performed better than the commercially available polyacrylamides but retained high amounts of water in the sediments. This comparison shows that polymers other than polyacrylamide may be used to treat iron ore tailings.

## 1. Introduction

The mineral extraction and ore processing industry has grown significantly over the last several years, raising concerns regarding its impact on the environment [1]. Tailings generated during mineral extraction are mainly made of water and fine particles that form a stable colloidal system and make water recycling difficult [2]. Even though the submicron particles in these colloidal suspensions may sediment by gravity, the process may take several years [3,4].

The growth in demand for mineral products is making the mining and processing of lower-grade ores inevitable, requiring more energy and resources. In this case, ore grinding and classification must be intensified to obtain the metal of interest, which generates more water and fine particles to be disposed of in tailings ponds [5,6,7,8].

A chemical compound, such as a polymer flocculant, is often added to mineral tailings before they are pumped into tailings ponds. Polymers ease the solid–liquid separation by bridging, neutralizing charges, or forming electrostatic patches with suspended particles [9].

Typically, mineral tailings are treated with polyacrylamide (PAM), which can be anionic, cationic, or non-ionic. PAM is widely used because it forms large flocs that settle quickly on the thickeners’ bottom. Unfortunately, PAM flocs retain a significant amount of water due to the high hydrophilicity of the amide and carboxylic groups on the PAM molecules. Many studies have shown that hydrophobic molecules can be incorporated into hydrophilic flocculants to address these limitations. Reis et al. [10] synthesized copolymers of polypropylene oxide macromonomers (PPO) and acrylamide with different hydrophobicities and molecular weights for dewatering oil sand tailings, comparing their separation efficiency with commercial anionic PAM. The authors found that the new PAM-PPO copolymers flocculated and dewatered tailings with high solid content (20 wt.%) more effectively in terms of turbidity, settling rate, and dewaterability. However, there is also a limit to how much PPO can be incorporated into the polymer before the hydrophobicity dominates and separation efficiency is adversely affected. Hripko et al. [11] synthesized PAM with poly(ethylene glycol) methyl ether methacrylate (PEOMA) and found that this polymer dewatered MFT more effectively than commercial polyacrylamide alone due to its hydrophobicity. An environmentally friendly PAM was proposed by Bazoubandi and Soares [12] by grafting acrylamide onto amylopectin, a natural polymer. As a result of amylopectin’s hydrophobicity, amylopectin-graft-polyacrylamide flocculants performed better than commercial PAMs in terms of dewaterability.

Although the main concern of PAM usage is water retention, the following disadvantages should also be addressed: (1) high molecular weight PAM flocculants are difficult to disperse in tailings with high solid content [13]; (2) PAM degrades slowly in water, which negatively impacts the environment [14,15]; (3) to enhance the particle-PAM bonding and improve separation efficiency, divalent cations are needed, resulting in chemical contamination and infeasibility of recycling water [16,17,18]; (4) acrylamide can be carcinogenic and have other toxic properties [13,19]. These disadvantages have increased the interest in replacing PAM with other flocculants that are more environmentally friendly.

The performance of polymer flocculants is strongly correlated with the chemical and mineral composition of tailings: polymers engineered to flocculate one tailing type may not work equally well with other types. Many studies have pointed out the drawbacks of PAM and the system-specific application characteristics of new flocculants, so that they can be modified or substituted to reduce their disadvantages and risks. However, there is still a surprising lack of publications on the dewatering of iron ore tailings. Xu et al. [20] used an anionic PAM in combination with chitosan-graft poly(acrylamide-dimethyl diallyl ammonium chloride) (Chi-g-P(AM-DMDAAC)) to flocculate a kaolinite suspension. The positively charged Chi-g-P(AM-DMDAAC) adsorbed on the suspended particles, easing the adsorption of anionic PAM, which accelerated the flocculation. Dubey et al. [21] investigated the settling behavior of iron tailings with a binary graft copolymer flocculant of starch (St), polyacrylamide (pAAm), and poly(sodium p-styrene sulfonate) (pSS) (St-g-(pAAm-co-pSS)), and compared it with four commercial flocculants, mostly composed of acrylamide and acrylate. Compared with commercial flocculants, the novel polymers had faster-settled rates and produced clearer supernatants.

In this study, we investigated how two commercial PAMs with different molecular weights flocculated and dewatered a 5 wt.% iron ore suspension. Our main objective was to understand why PAM continues to be widely used to treat iron ore and other mineral tailings. We also tested two new polymers, developed to treat oil sand tailings, which are chemically and mineralogically distinct from iron ore tailings. The flocculation/dewatering performance of the polymers was measured using multiple light scattering, initial settling rate (ISR), supernatant turbidity, capillary suction time (CST), and solid contents of the sediments. The higher molecular weight PAM performed a little better than the low molecular weight PAM, but neither could clarify the tailings supernatant. The polymers developed for oil sands application did unquestionably better than polyacrylamide. These findings show that polymers other than PAM are viable alternatives for the flocculation and dewatering of iron ore tailings.

## 2. Materials and Methods

### 2.1. Materials

**Figure 1:**
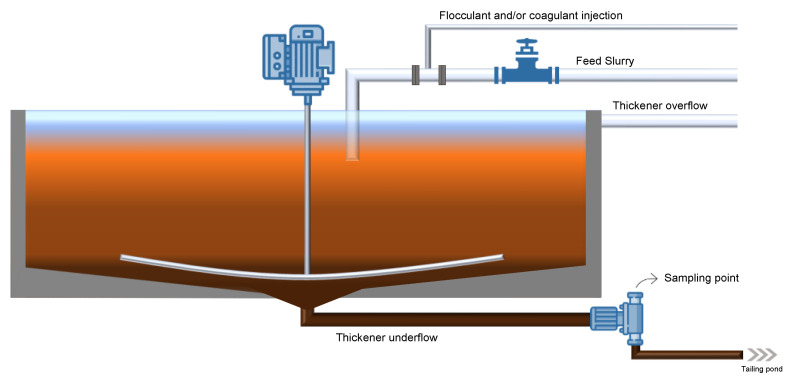
Thickener scheme.

The iron ore tailings samples were collected in the thickener underflow stream, immediately before being disposed into the Vargem Grande pond (Nova Lima, Minas Gerais, Brazil), as shown in Figure 1. Two commercial non-ionic polyacrylamides were used as flocculants: (1) PAM1 was obtained from Sigma-Aldrich (M_w_ = 5–6 MDa) (Oakville, ON, Canada) and is widely used in laboratory-scale flocculation, and (2) PAM2 was obtained from Kemira Chemicals (M_w_ = 1–20 MDa) (Brantford, ON, Canada) and is commonly used to treat industrial mineral tailings. Sodium hydroxide (NaOH) (≥98%), sodium sulphate (Na_2_SO_4_) (≥99%), and acetic acid (CH_3_CO_2_H) (≥99%) was purchased from Sigma-Aldrich (Oakville, ON, Canada). Poly((vinylbenzyl)trimethylammonium chloride) (PVB) and a partially hydrolyzed poly (methyl acrylate) grafted onto ethylene-propylene-diene copolymer backbones (EPDM-g-HPMA) were synthesized in our lab and used as flocculants. The synthesis and characterization of these polymers were reported in our group’s previous work on oil sand tailing flocculation [22,23].

### 2.2. Methods

#### 2.2.1. Handling and Drying the Tailing Samples

We dried the tailings samples (to make them easier to store and transport) according to the procedure: (1) drying in an oven at 60 °C for 72 h; (2) sieving in a 600 micron sieve to break the clods formed during drying; (3) homogenizing the dried tailings (using the long pile quartering method) by spreading the dry clods back and forth on a clean surface, making a triangular cross-section pile of about 5 m in length; (4) removing the less homogeneous ends of the pile and spreading them over the pile; and (5) fractionating the pile into 600 g samples. Later, we used these dried samples to prepare colloidal suspensions for the flocculation studies.

#### 2.2.2. Suspension Preparation for Flocculation Tests

**Figure 2:**
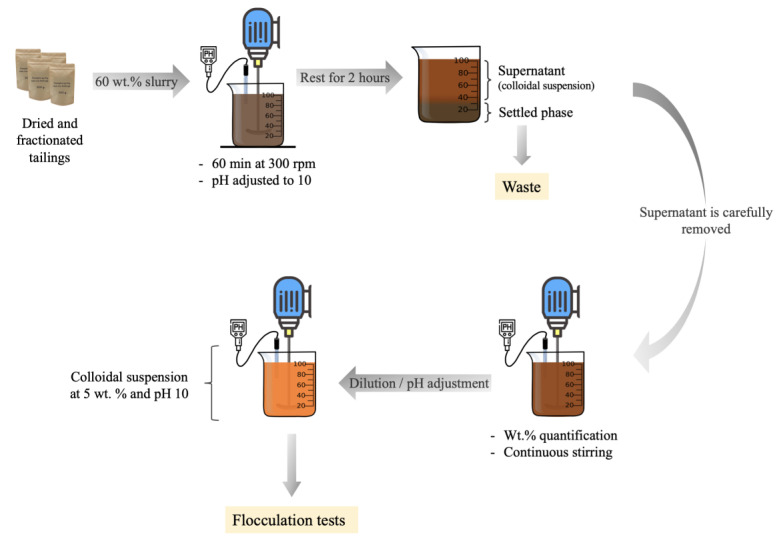
Schematic of flocculation media 5 wt.% preparation.

We prepared suspensions for the flocculation tests by mixing the dry tailings with deionized water to make a slurry with 60 wt.% solids, which is similar to the concentration in the thickener underflow stream. First, we added water to the solids and stirred the mixture for 60 min at 300 rpm. During the mixing, we adjusted the pH to 10 by adding 0.5 M NaOH. Next, we allowed the mixture to rest for 2 h to allow the coarse particles to settle, leaving a supernatant colloidal suspension of fine particles. Then, we carefully transferred the supernatant to another recipient where it was stirred at 300 rpm and its pH was adjusted to 10 using the same NaOH solution. Finally, we diluted the solids content to 5 wt.%, using deionized water. We calculated the amount of water that needed to be added to obtain the 5 wt.% colloidal suspensions after determining the solid contents of the supernatant by placing 1 mL aliquots in an MB45 moisture analyzer (in triplicate). A schematic for the preparation of the flocculation suspension from the dried tailings is shown in Figure 2.

#### 2.2.3. Polymer Characterization

Fourier transform infrared spectroscopy (FTIR) was used to confirm the presence of the characteristic groups of the non-ionic PAM by attenuated total reflection (ATR) using an Agilent Cary 600 FTIR spectrometer. The data were collected in the range from 400 to 4000 cm^−1^. The weight average molecular weight (*M_w_*) of all polymers was characterized by gel permeation chromatography (GPC) (1260 Infinity Multi-Detector GPC/SEC System, Agilent Technologies, Santa Clara, CA, USA) coupled with three detectors: viscosity, refractive index, and light scattering. Two columns (PL aquagel-OH MIXED-H 8 µm, 300 × 7.5 mm) connected in series were used to obtain a better resolution and increase the instrument’s detection range. Water containing 0.15 M Na_2_SO_4_ and 0.05 M acetic acid was used as a mobile phase for the analysis. The characterization PVB and EPDM-g-HPMA were previously reported by our group [22,23].

#### 2.2.4. Tailings Characterization

The solids (wt.%) content was determined gravimetrically, by heating approximately 1 g of the sample at 105 °C until all liquid was evaporated and a constant weight was obtained. The difference between the initial and final masses was used to calculate the solids content. All samples were analyzed in triplicate. The particle size distribution (PSD) of the tailing was obtained using a Mastersizer X particle size analyzer (Malvern Panalytical, Malvern, UK) in a range from 1 to 600 μm. The PSD of the dried samples was measured and compared with that of the tailings as received to identify any differences in particle size after drying and quartering. Because the Mastersizer X cannot analyze particles with sizes in the colloidal spectrum, a Zetasizer Nano ZS90 (Malvern Instruments, Malvern, UK) was used to determine the PSD of the colloidal suspensions. All samples were diluted to 1:100 using deionized water and the pH was adjusted to 10 by adding a 0.5 M NaOH solution.

X-ray powder diffraction (XRD) (Rigaku—Miniflex^®^, Tokyo, Japan) and X-ray fluorescence (XRF) (Malvern Panalytical-Zetium) were used to characterize the mineral phase and chemical composition, respectively, of the settled and colloidal phases from the 60 wt.% slurries. The XRD patterns were collected using Cu Kα radiation (45 kV, 40 mA, and λ = 1.5418 Å) at a scanning angle (2θ) from 2.5° to 80° and time per step of 200 s. The XRD patterns were analyzed with the X’Pert HighScore software. For the XRF analysis, the loss to fire (LF) was performed at 1020 °C for 2 h. The morphology and chemical composition of a single particle of the tailing samples was studied by scanning electron microscopy coupled with spectroscopy of dispersion energy (SEM-EDS) (JEOL JSM-7401F).

The ion composition of the aqueous phase of 5 wt.% colloidal suspension was determined by an inductively coupled plasma optical emission spectrometer, Spectro Arcos ICP-OES (Spectro Analytical Instruments GmbH, Kleve, Germany). The ions analyzed were Al, Ca, Fe, K, Mg, Mn, Na, Ni, Ti, and Zn. The aqueous phase was separated from the suspended solids using a 0.45 μm PTFE syringe filter. To ensure maximum reproducibility of the real tailing conditions, we assumed the chemical composition of the processing water (provided by the mining company) to be the same as in the tailing pond.

#### 2.2.5. Flocculation Tests

We varied the PAM dosage from 500 to 5000 ppm (grams of polymer per ton of suspended solids) to better demonstrate the economic viability of the flocculant. We also tested a dosage of 1000 ppm of two new polymers designed for treating oil sand tailings, poly(vinylbenzyl)trimethylammonium chloride (PVB), and partially hydrolyzed poly(methyl acrylate) grafted onto ethylene-propylene-diene copolymer backbones (EPDM-g-HPMA), to find out whether polymers designed to treat different tailings could be viable alternatives to PAM. The flocculation tests were all conducted at pH 10, reflecting the tailings’ pH environment.

##### Multiple Light Scattering Analysis

The settling behavior and dewatering efficiency of the tailings using different polyacrylamide concentrations were studied using an optical analyzer (Turbiscan Lab—Formulation, Toulouse, France) that relies on the multiple scattering behavior of light. A pulsed infrared laser (850 nm wavelength) moves vertically (up and down) along with a flat-bottomed cylindrical glass cell containing the fluctuating sample. Light transmitted and backscattered can both be measured using two detectors. Transmission and backscattering data acquisition are performed every 40 µm along the sample [24,25,26].

Each test was performed by placing 22 g of the 5 wt.% colloidal suspension into the sample vial, followed by the addition of the desired volume of the stock polymer solution. The mixture was immediately stirred for precisely 30 s at 300 rpm using a Vortex mixer (Stuart, SA8) and then fitted to the equipment, standing the readings. Data acquisition was set for every 5 s for 24 h.

The Turbiscan^®^ stability index (TSI) was calculated from the backscattering data with TurbiSoft 2.0.0.33 software. The TSI is a dimensionless parameter proposed by the equipment manufacturer to study the effects on suspension stability over time. It is calculated based on the sum of all variations in TS and BS that are directly related to the destabilization of the suspension, as shown in Equation (1). Therefore, the larger the TSI value, the lower the stability of the medium [27].

(1) $$TSI(t)=\frac{1}{N_{h}}\sum_{t_{i}=1}^{t_{max}}\sum_{z_{i}=z_{min}}^{z_{max}}\left|BST(t_{i},z_{i})-BST(t_{i-1},z_{i})\right|$$

In Equation (1), $t_{max}$ is the measurement point corresponding to the time t at which the TSI is calculated, $z_{min}$ and $z_{max}$ are the lower and upper selected height limits, $N_{h}=(z_{max}-z_{min})/∆h$ is the number of height positions in the designated zone of the scan, and BST is the considered signal (BS if I < 0.2%, T otherwise).

Naked eye visualization is commonly used in flocculation studies. In general, the visual appearance varies considerably with high flocculant dosages, and it cannot be detected at lower concentrations. To better analyze the data, we used correlation between visual observation and the Turbidity Stability Index (TSI), which reflects the intensity of destabilization. According to their model, TSI values are associated with categories from A+ to D [27]. TSI below 0.5 indicates negligible destabilization (A+), while 0.5–1 means early-stage destabilization (A), yet neither is visually noticeable. Destabilization begins between 1 and 3 TSI (B), and significant changes occur between 3 and 10 TSI (C); both changes are mostly non-visual. In the case of a TSI above 10 (D), extensive, typically visible destabilization is expected.

##### Initial Settling Rate (ISR)

A sample of 100 g of the prepared tailing suspension (5% by mass) was placed in a beaker and allowed to mix for 1 min at 500 rpm with a mechanical stirrer coupled with a 3-bladed propeller stirrer (IKA—R-1388). Then, the polymer solution was added (always in the same spot) and stirred for another 1 min and 500 rpm, followed by 2 min at 280 rpm. Interactions between polymers and suspended particles are favored at high stirring rates, while polymer/particles form larger flocs at low stirring rates. Finally, the suspension was rapidly transferred to a 100 mL graduated cylinder, where the mudline height was recorded as a function of time for 10 min. Initial settling rate (ISR) was calculated from the slope of the h/H versus time plot, where h is the mudline height, and H is the total suspension height at the start of flocculation. The slope was determined for the first 2 min of flocculation.

##### Capillary Suction Time (CST)

Capillary suction time (CST) measures the time required for water to travel between two electrodes in a cellulose filter paper. The rate at which solvent flows through the porous surface is proportional to the suspension dewaterability. All readings were done by pouring 3 mL of the sedimented phase (24 h after adding the flocculant) in an open cylinder in contact with the CST filter paper 7 × 9 cm (Triton Electronics, Essex, UK) and using a Type 319 Multi-Purpose CST apparatus (Triton Electronics, Essex, UK). Two electrodes, placed 0.5 cm from each other, measured the time taken by the water to travel radially between them.

##### Zeta Potential Measurements

The zeta potential of 5 wt.% colloidal suspensions without flocculant and after 24-h flocculant addition was determined by electrophoretic light scattering using a Zetasizer Nano ZS90 (Malvern Instruments, Malvern, UK). Samples were diluted to 1:100 using deionized water at pH 10, adjusted with a 0.1 M NaOH solution. The samples were permanently removed from the same spot. The temperature was set at 25 °C for all runs. The effect of pH ranging from 2 to 10 on the zeta potential of the 5 wt.% colloidal suspensions was also studied. The pH adjustment was made by adding 0.1 M NaOH or 0.1 M HCl solutions.

## 3. Results and Discussion

### 3.1. Polymer Characterization

**Figure 3:**
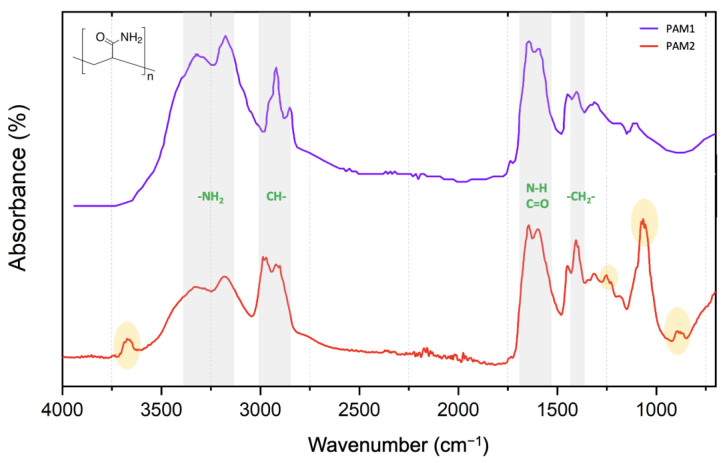
FTIR spectra of polyacrylamide samples PAM1 and PAM2.

Figure 3 compares the FTIR spectra of the commercial polyacrylamide samples PAM1 and PAM2. The double peak in the 3100 and 3400 cm^−1^ range confirms the presence of NH_2_ groups, while the peak at 1650 cm^−1^ is related to the C=O and N-H bonds. The peaks at about 2900 cm^−1^ and 1400 cm^−1^ are due to the presence of C-H and C-H_2_ groups, respectively [28].

The FTIR spectra of sample PAM2 also detected a few peaks that were absent from the spectra of sample PAM1 (orange oval marks in Figure 3). Sample PAM1 is made only for research purposes but sample PAM2 is a commercial flocculant developed for the mining industry. Therefore, the extra peaks in sample PAM2 are probably related to additives—which are considered intellectual property and not disclosed by the manufacturer—which may explain its better performance, as we will discuss below. Other works also reported similar considerations about commercial polymers for industrial flocculation purposes [29,30,31].

The weight average molecular weights, *M_w_*, and dispersities, *Đ*, of samples PAM1 and PAM2 were measured by GPC and are listed in Table 1.

The *M_w_* measured for sample PAM1 falls within the range reported by the manufacturer (*M_w_* = 5–6 MDa), but no data were reported for *Đ*. The low *Đ* value indicates that the polymer is likely made by controlled free-radical polymerization under uniform synthesis conditions. The *M_w_* value measured for sample PAM2 was also included within the broad range provided in manufacturer’s datasheet (*M_w_* = 1–20 MD) for this brand of flocculants. Abu-Zreig et al. [32] reported *M_w_* = 12–15 MDa for PAM samples from the same family.

### 3.2. Tailing Characterization

#### 3.2.1. Solid Content

The solids content of the sedimented phase of the tailings sample (as received) was 87.3 ± 0.4 wt.%. This value was higher than in the thickener underflow stream (about 60%), which is the most concentrated fraction of the thickening stage. The solids content of the sample was higher because the tailings sample settled for a few weeks and part of the supernatant water (clarified phase) was removed to reduce its volume. However, the higher solids content had no impact on this work since the concentration was adjusted to 5 wt.% for all tests.

A sample of the supernatant of the received tailings was reserved to determine its solid content. Then, we dried the tailings samples and used it to prepare the flocculation media by mixing it with water to form a 60 wt.% tailing and adjusting its pH to 10 to reproduce the thickener underflow concentration. After the prepared 60 wt.% tailing settled for 2 h, two phases were formed: a coarse particle layer at the bottom (sedimented phase) and a colloidal suspension at the top (suspended phase). We separated both phases and measured their solids content. We diluted the suspended colloidal phase to 5 wt.% and adjusted its pH again to 10 to match the value in the tailings pond. The concentration of 5 wt.% is commonly adopted in the literature to investigate polymer flocculants [33,34,35,36].

The solids contents of the tailing supernatant (as received) and of the sedimented and suspended phase from the prepared 60 wt.% tailing is shown in Table 2.

The solid content of the sedimented phase of the 60 wt.% tailing was slightly smaller, compared to the same phase of the received material (87.3 ± 0.4 wt.%). This result was unexpected, as the prepared 60 wt.% tailing aims to reproduce the tailings samples collected in the thickener’s underflow. This difference was attributed to the shorter settling time (2 h) and no removal of the clarified phase for the prepared suspension.

Regarding the suspended colloidal phases, the solid content was smaller for the received tailings, because they were stored in the laboratory for several weeks before use, leading to more particles to settle naturally.

#### 3.2.2. Particle Size Distribution

**Figure 4:**
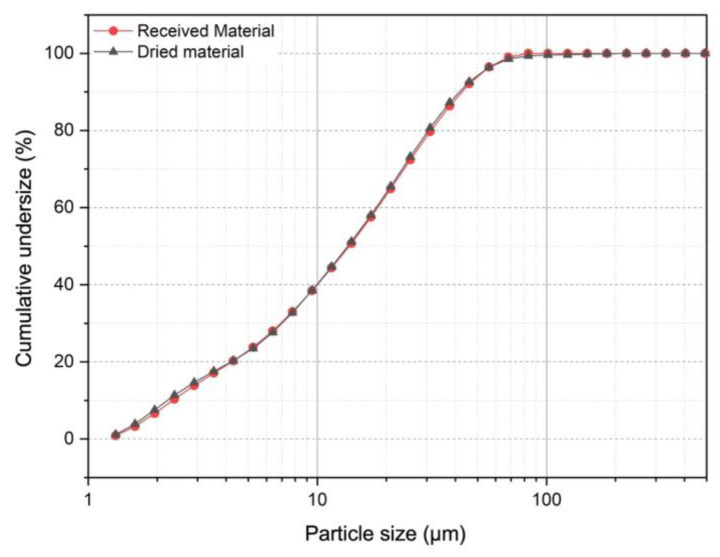
Volume-based cumulative PSD of the received (red curve) and dried (blue curve) tailings.

Figure 4 shows that the volume-based cumulative particle size distributions of the received and dried tailings are nearly the same, proving that the drying procedure did not change the particle sizes of the tailings sample. The D10, D50, and D90 of both tailings were approximately 2.0, 13.6, and 41.9 µm, respectively. Colloid suspensions are most likely to form with particles smaller than 2 m and are found in 10% of the tailing sample.

**Figure 5:**
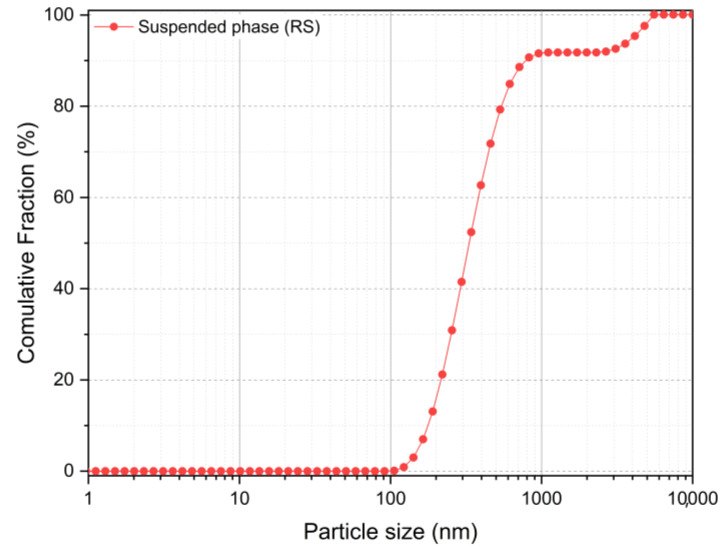
Volume-based cumulative PSD of the suspended colloidal phase (supernatant) from the prepared 60 wt.% slurry.

The PSD of the 5 wt.% suspension used in all flocculation tests is shown in Figure 5. More than 90% of the sample is composed of particles smaller than 1 µm.

#### 3.2.3. Chemical and Mineralogical Composition

**Figure 6:**
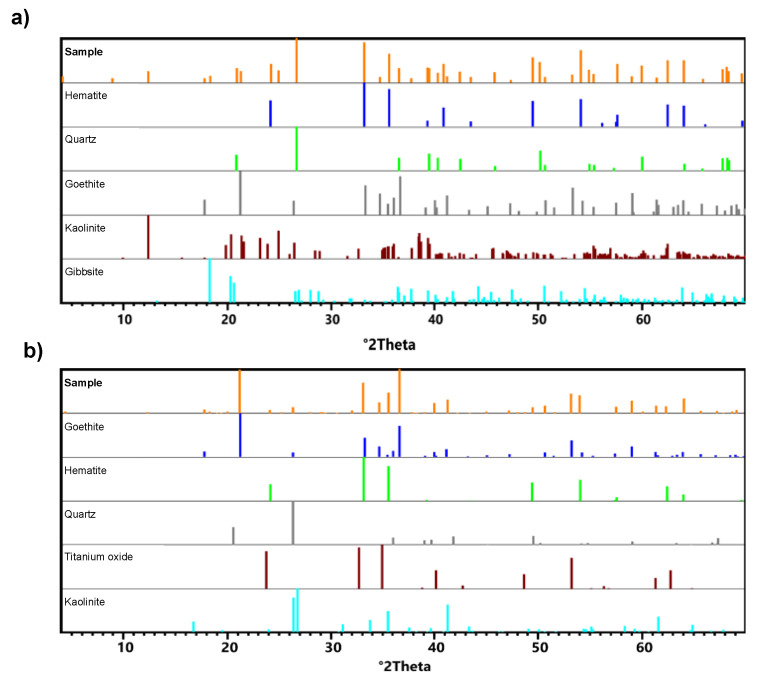
XRD patterns of the sediment (**a**) and colloidal suspension (**b**) from the 60 wt.% slurry.

The chemical composition (measured by XRF) and mineral phase identification (measured by XRD) for sedimented and suspended phases formed in the prepared 60 wt.% tailing are shown in Table 3 and Figure 6, respectively.

Both the suspended and sedimented phase were mainly composed of iron oxides, SiO_2_, and Al_2_O_3_. Iron was the predominant compound, followed by SiO_2_ and Al_2_O_3_ in the sediment, and Al_2_O_3_ and SiO_2_ in the colloidal suspension. Comparing the sediment with the colloidal suspension phase, SiO_2_ decreased by 88% and Al_2_O_3_ increased by 207%. These oxides correspond to hematite (α–Fe_2_O_3_), quartz (SiO_2_), goethite (α–Fe^3+^O(OH)), kaolinite (Al_2_Si_2_O_5_(OH)_4_), and gibbsite (Al(OH)_3_). Titanium oxide (TiO_2_) was only identified in the colloidal suspension. Hematite and quartz were the most abundant minerals in the sedimented coarse particles, while goethite and hematite represented the colloidal suspension. These findings were consistent with a comparable study reported by Nanda and Mandre (2022) [37]. The lower SiO_2_ concentration in the colloidal supernatant suggests that SiO_2_ particles are bigger and, therefore, prominent in the sediment. In addition, Gomes (2011) [38] also observed higher Al_2_O_3_ content in the fine fractions of iron ore tailings and concluded that they were associated with the silt-clay portion of the tailings. According to Andrade (2014) [39], the concentration of Al_2_O_3_ in iron ore tailings is determined by the geology of the extraction region, which may be rich in phyllites with aluminosilicates.

#### 3.2.4. Scanning Electron Microscopy

**Figure 7:**
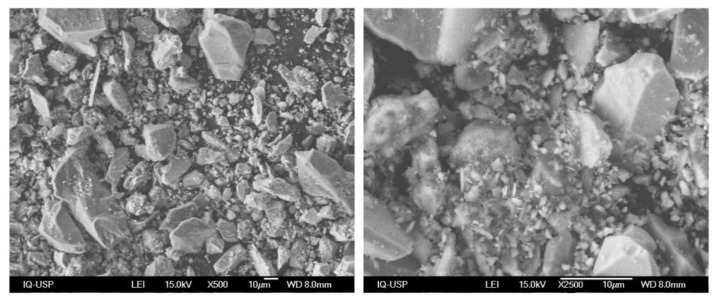
SEM images of the received tailings.

The surface morphology of the dried tailings was studied by scanning electron microscopy (SEM), as shown in Figure 7. The tailings were polydisperse, with the predominance of angular and granular particles. The polydispersity is consistent with the PSD of the material (see Figure 4 and Figure 5).

**Figure 8:**
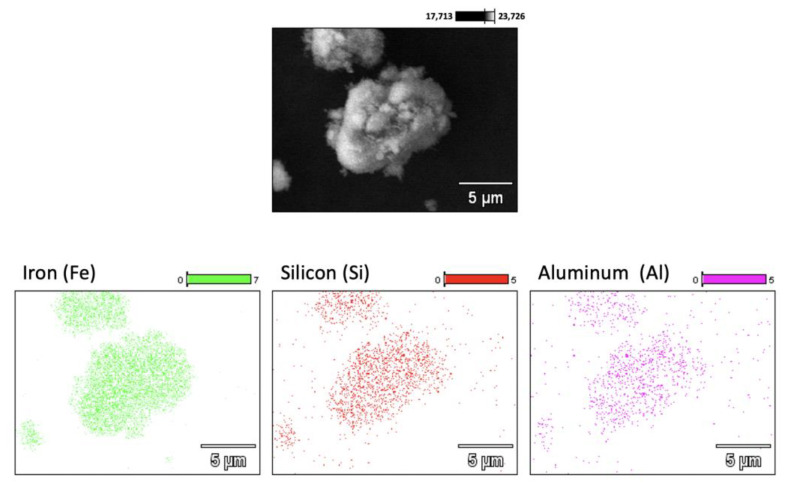
EDS elemental mapping of a single particle of the dry tailings.

The elemental mapping of a single particle surface of the dry tailing was done by energy dispersive spectroscopy (EDS), as shown in Figure 8. The color density of the images is proportional to the amount of the element found on the particle surface. Fe, Si, and Al were detected along the particle surface. These findings are consistent with the chemical and mineralogical results. The presence of different elements in a single particle implies that the extracted rocks consist of aggregates of different minerals, segregated into distinct phases. Among the detected elements, iron comes from hematite and goethite iron-containing minerals, silicon was found in quartz and kaolinite, while aluminum was detected from the kaolinite and gibbsite mineral phases.

#### 3.2.5. Chemical Composition of the Aqueous Phase

**Figure 9:**
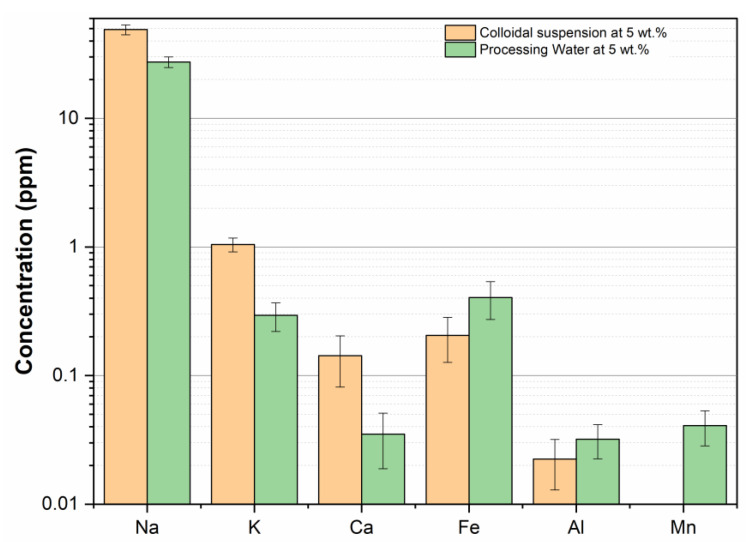
Chemical composition of the aqueous phase of the prepared flocculation media and the processing water at 5 wt.%.

Because the zeta potential and colloidal stability depend strongly on the system composition, one must prepare flocculation media that resemble tailings as closely as possible [40,41]. Since the composition of tailing varies considerably along the pond, the process water composition (provided by the tailings supplier) was assumed to be similar to the composition in the tailing pond. The concentration of the ions in the process water was converted to a 5 wt.% suspension (same as the suspension used in the flocculation tests), by assuming the solids content of the tailings pond was the same as for the 60 wt.% slurries. The chemical composition of the process water and of the aqueous phase of the 5 wt.% colloidal suspensions (determined by ICP-OES) are shown in Figure 9.

Sodium, potassium, and calcium ions were more abundant in the 5 wt.% colloidal suspensions. Grilo et al. [42] studied the effect of the concentration of different salts on the zeta potential of iron mining tailings from the Fundão dam in Minas Gerais (Brazil). According to the authors, by increasing 20 times (from 0.5 to 1.0 mM) the concentration of NaCl, KCl, and CaCl_2_, the zeta potential increases by 5, 4, and 46%, respectively. It was expected that monovalent ions, such as sodium and potassium, do not affect the zeta potential. However, the opposite is observed for divalent cations. Higher the ion valency, the higher the neutralization and compression effect on the EDL, favoring particles’ destabilization. We decided not to change the calcium concentration since a higher content of this cation enhances the flocculation process, which is the objective of this work. In addition, the concentration was considered slightly different, if the experimental error was considered.

Iron and aluminum concentration was considered the same for both, according to the experimental errors. Aluminum was the less abundant among all analyzed elements for the 5 wt.% colloidal suspensions, probably because this element was in the ore solid form. This result is consistent with the chemical quantification of the solid colloidal phase, where Al_2_O_3_ had the second-highest concentration. Because manganese was detected only in the processing water, it was added (with MnSO_4_) to the prepared suspension to compensate for possible the effects of this polyvalent ion on the system’s zeta potential.

#### 3.2.6. Zeta Potential Measurements

**Figure 10:**
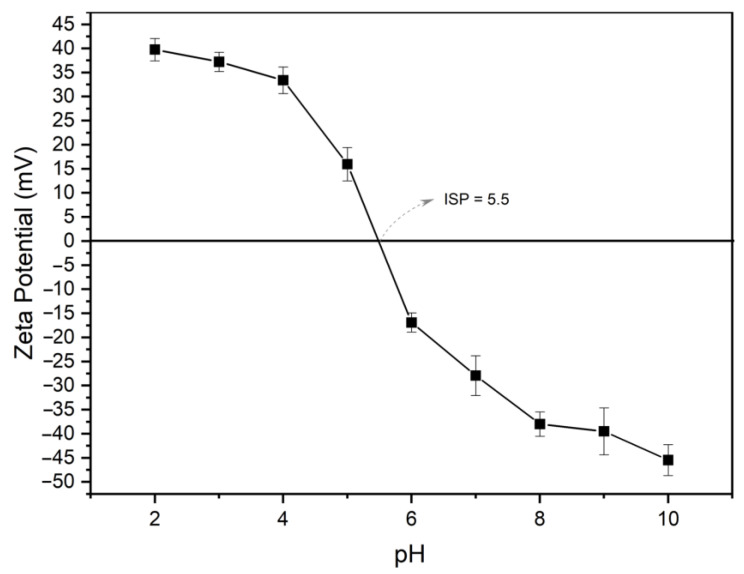
Zeta potential measurements of the 5 wt.% prepared suspensions at different pH (2 to 10).

The effect of pH ranging from 2 to 10 on the zeta potential of the 5 wt.% prepared suspensions was studied by the addition of 0.1 M NaOH or 0.1 M HCl solution. The results are shown in Figure 10.

The isoelectric point (IEP) was found to be pH 5.5. Since each mineral phase of the tailings has its own IEP (ranging from 1.8 to 9.5), the ISP of 5.5 seems reasonable if the proportion among them is taken into account [43,44,45,46,47]. Furthermore, the mineral surface protonates at low pH levels (below the IEP), which produces a positive zeta potential. In the same way, mineral surfaces are deprotonated at high pH (above the IEP). Finally, the particle state is neutral at a high pH [48].

### 3.3. Flocculation Tests

We flocculated 5 wt.% iron ore tailing suspensions using two commercial non-ionic polyacrylamides, PAM1 and PAM2, investigating the effect of dosages from 500 to 5000 ppm. Flocculant performance was investigated by multiple light scattering, solid contents, zeta potential, initial sedimentation rate (ISR), and capillary suction time (CST).

#### 3.3.1. Effect on Initial Settling Rate (ISR)

**Figure 11:**
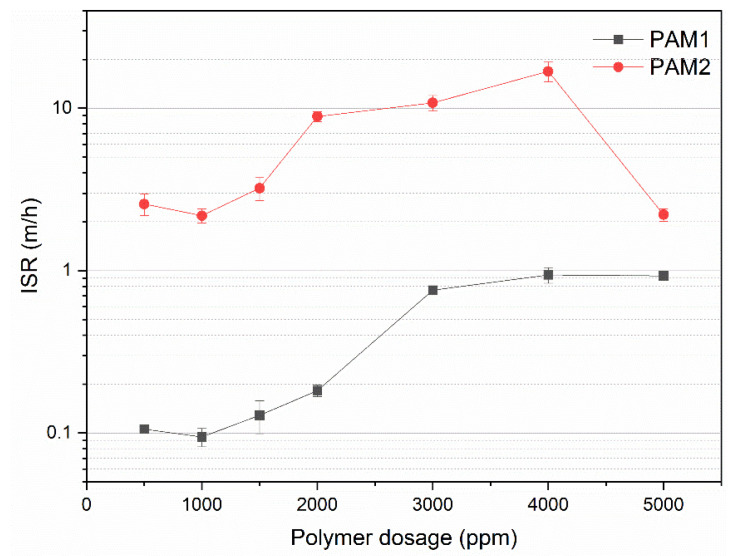
Initial settling rate for flocculation with PAM1 and PAM2 at different polymer dosages.

Figure 11 shows the ISR for flocculation with PAM1 and PAM2 at different polymer dosages. For most conventional thickeners, the settling rate is considered low when ISR < 5 m/h and high when ISR ≥ 20 m/h [49,50]. For PAM2, the ISR exhibited the typical behavior of polymer flocculants, increasing with dosage until reaching a maximum value, and decreasing for higher dosages. This effect is explained by the increase in polymer–particle bridges which leads to bigger flocs that settle faster. After an optimal dosage, typically covering 50% of the particle surface, the polymer molecules start hindering bridge formation (overdosing), which results in smaller flocs that settle more slowly. In addition, steric repulsions between polymer chains may stabilize the suspension [34,51,52,53].

A similar effect was observed for PAM1, where ISR increased up to 3000 ppm, but unlike PAM2, reached a plateau. Since PAM1 has a lower *M_w_* and a much narrower molecular weight distribution, it is not surprising that the maximum ISR occurs at a different polymer dosage. Moreover, for the same dosage of both polymers, the ISR for PAM2 was always higher, which may also be attributed to the higher *M_w_* of PAM2. Increasing *M_w_* is a strategy for improving flocculation efficiency, especially in the case of non-ionic polymers, in which there is no charge in the polymer chains [13,54].

#### 3.3.2. Effect on Transmission (%) and Backscattering (%)

**Figure 12:**
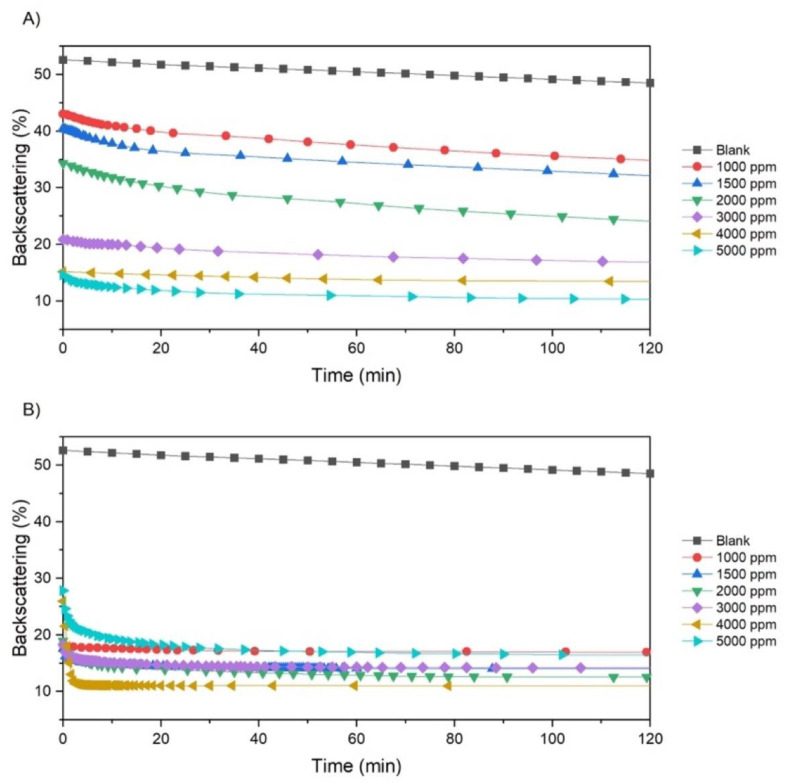
Backscattering as a function of time in the clarification zone for PAM1 (**A**) and PAM2 (**B**) at different dosages.

The floc formation and settling profile was investigated by multiple light scattering. After the polymer addition, the solids concentration decreases from top to bottom, forming a clarification zone at the top and a thickening zone at the bottom. The change in light backscattering percentage in the clarification zone was used to evaluate the flocculation dynamics and tells how fast the polymers form flocs that precipitate to the bottom of the vial. Figure 12 compares the dynamic backscattering values for PAM1 and PAM2 at several dosages. Because no significant change in backscattering was observed after 2 h, only this time range was considered. This analysis will not take into account results for 500 ppm, as they were not reproducible due to high turbidity.

All BS% values decreased with increasing PAM1 dosage, reaching a minimum of 11% at 5000 ppm. At this concentration, at about 2 h of flocculation, BS% tends to approach a steady state. For the blank suspension (without flocculant), it took about 60 days to reach the same conditions. Although a significant reduction was obtained, 2 h still seems industrially impractical. Furthermore, a BS of 11% represents very poor clarification efficiency.

PAM2 had a smaller BS% for all dosages. BS% approached a steady state at 50, 50, 47, 40, 6, and 50 min for 1000, 1500, 2000, 3000, 4000, and 5000 ppm, respectively. At 4000 ppm, the system quickly tends to the steady state and a minimum BS% of approximately 11%. By increasing the dosage to 5000 ppm, the BS% values increased and reached a minimum of 16%. This result seems to be caused by polymer overdosing, forming smaller flocs that suffer steric repulsion. The higher *M_w_* of PAM2 and the possible presence of additives are likely responsible for improving the separation efficiency.

**Figure 13:**
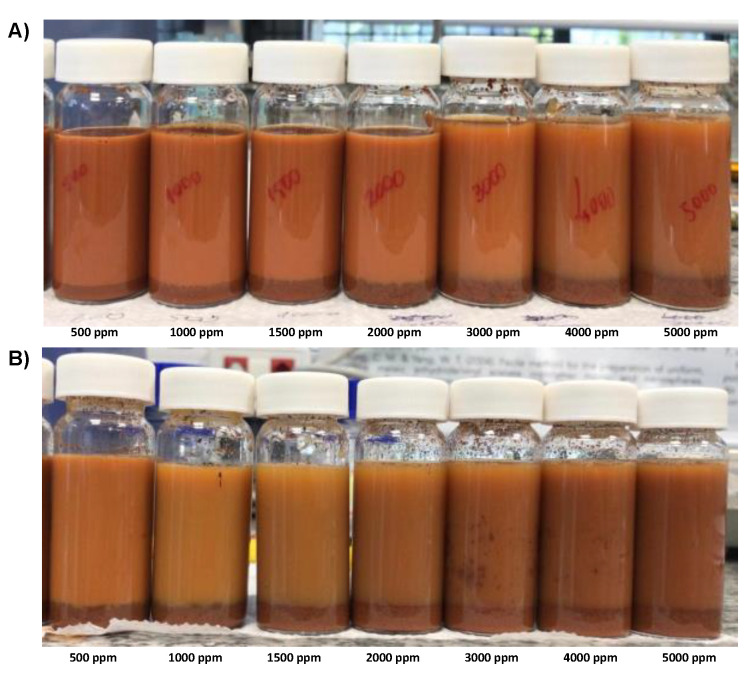
Flocculation samples after adding different dosages of PAM1 (**A**) and PAM 2 (**B**).

Ideally, BS% should approach zero at a steady state, indicating that no particles remain suspended in the supernatant. However, among all conditions we tested, the minimum BS% of 11% was not enough to clarify the supernatant. This conclusion is supported by the pictures shown in Figure 13.

**Figure 14:**
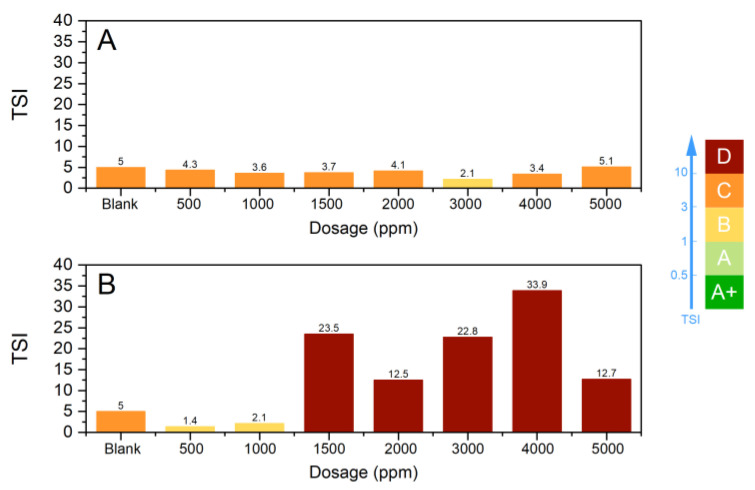
TSI of the whole sample with PAM1 (**A**) and PAM2 (**B**) as flocculants and different dosages.

The turbiscan stability indices (TSI), calculated with the TurbiSoft software, are compared in Figure 14. We also used the TSI scale, proposed by the manufacturer, to discuss the system stability based on the correlation of the TSI values with visual methods [27].

The lower the TSI, the more stable the suspension. The TSI for PAM1 varied from 2.1 to 5.1. According to the TSI scale, from 3 to 10, the TSI falls into group C, in which there is a certain degree of destabilization detected both experimentally and with the naked eye. At this stage, the flocs may be formed with a broad particle size distribution and precipitate poorly. Both phenomena were visualized during the experiment, even at the beginning of flocculation (as shown in Figure 14A).

As expected, PAM2 performed better than PAM1 (Figure 14B). Above 1500 ppm dosages, all TSIs belong to group D, which corresponds to considerable medium destabilization, significant sedimentation, phase separation, and widespread change in particle size or color. After 4000 ppm, there is a reduction in TSI, indicating the suspension stabilization are being restored. This result corroborates to the steric repulsion effect due to the polymer excess.

#### 3.3.3. Solid Content

**Figure 15:**
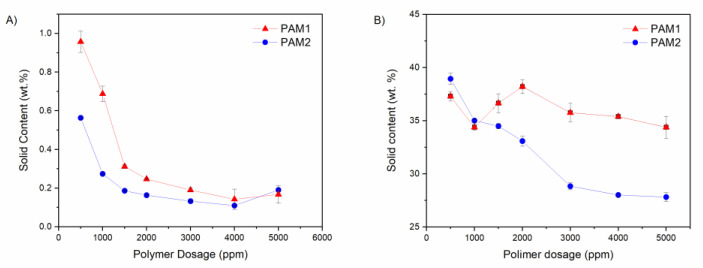
Solid content of the supernatant (**A**) and sedimented phase (**B**) after 24 h flocculation with PAM1 and PAM2.

The solids contents of the supernatant and sedimented phase after 24 h flocculation are shown in Figure 15.

Higher dosages of PAM1 and PAM2 reduced the solids content of the supernatant (shown in Figure 15A). For all dosages, the values were higher for PAM1. This finding agrees with our previous observation that PAM1 is less efficient than PAM2: the lower the *M_w_*, the more particles are left suspended in the supernatant. In addition, a slight increase in the solids content of the supernatant was observed when the dosage of PAM was 5000 ppm. This result is consistent with the BS% discussion, where an increase of this parameter was observed at 5000 ppm.

Regarding the supernatant, PAM1 performed worse than PAM2. On the other hand, for the sediment, the opposite was observed (Figure 15B). This was attributed to the higher hydrophilicity and Mw of PAM2, which promotes a better settling of the suspended fine particles, and retains more water in the floc’s interstices, due to the predominance of h-bonding.

#### 3.3.4. Capillary Suction Time (CST)

**Figure 16:**
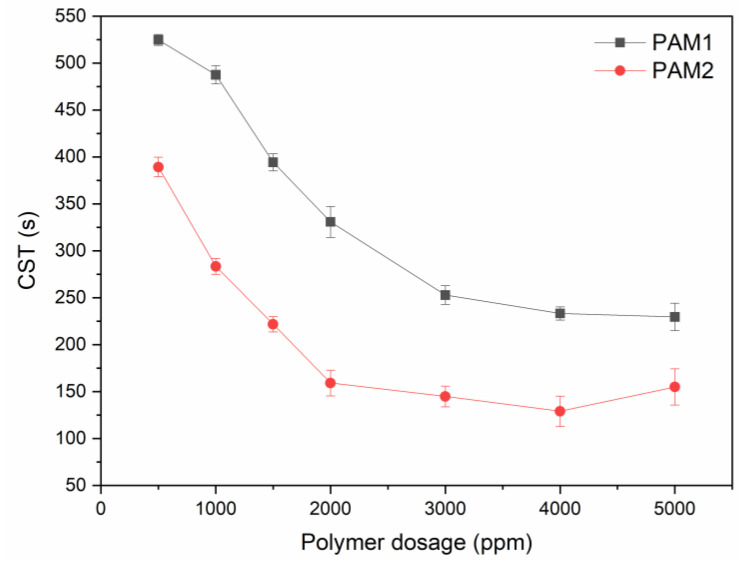
CST for PAM1 and PAM1 at different dosages.

Figure 16 shows the CST of the sediments formed after 24 h of flocculation under different dosages of PAM1 and PAM2.

Although most relevant studies with CST focus on oil sand tailings, this parameter was useful to predict the dewaterability of the iron ore tailings under flocculation with PAM. As a reference, we used the protocol proposed by the Oil Sands Tailings Consortium (OSTC), where the sediment has good dewaterability if the CST is less than 100 s. CST decreased with increasing dosage for PAM1 and PAM2. As molecular weight increases, aggregates are expected to be larger and more compact, retaining less water, and resulting in lower CSTs. This decrease in CST is reversed when polymer overdosing is reached (visible by the increase for PAM2 after 4000 ppm). This polymer excess effect was explained based on the hydrophilic character of PAM [55]. After a certain concentration, water retention is favored by intermolecular interactions, both inside the formed floc and residual polymer. In other words, water tends to flow slowly due to the dominance of stronger interactions with the water molecule from the hydrogen bonds formed with the polymer’s amide group [56,57]. For PAM1, this trend was very subtle if the experimental error was considered. This result corroborates previous discussions in which for lower Mw, higher dosages are necessary to achieve saturation. Similar results were observed by Lee and Liu [56] where higher molecular weight resulted in optimal CST at lower dosages. The lowest CST among all tested conditions was of 129 s (for PAM2 at 4000 ppm), which was not in the range adopted as a parameter for good dewatering efficiency.

#### 3.3.5. Zeta Potential Measurement

**Figure 17:**
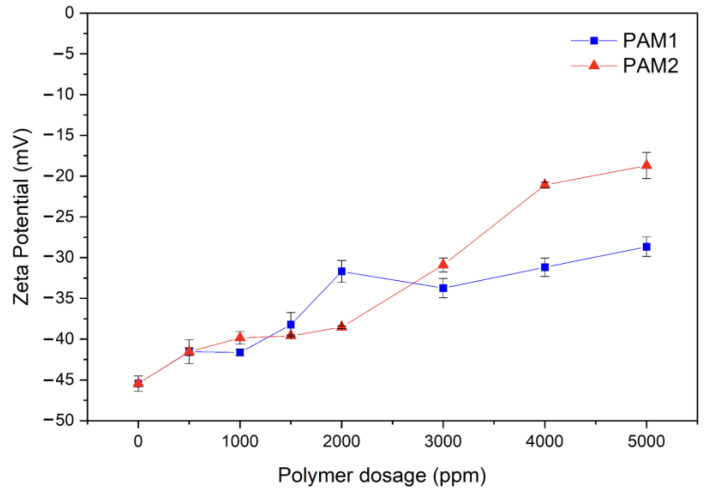
Zeta potential of the supernatant at different flocculant dosages of PAM1 and PAM2 and after flocculating for 24 h.

A polymer’s flocculation effectiveness depends on its size, structure, charge, and the way it interacts with suspended particles. In this study, a 5 wt.% tailing suspension with pH 10 was flocculated with two non-ionic polyacrylamides (PAMs) of different molecular weights (Mw_PAM2_ > Mw_PAM1_). Zeta potentials of the supernatant after 24 h flocculation with different dosages of PAM1 and PAM2 were determined to study the effect of these polymers on the surface charge of the suspended particles, and the results are shown in Figure 17.

Zeta potentials indicate whether colloidal systems are stable. Those with high absolute values (negative or positive) are stable, while those with low absolute values are more likely to coagulate or flocculate. The zeta potential for the colloidal suspension (blank) was found to be −45.45 mV, indicating that the particles are stable and negatively charged. With the addition of the PAMs, the zeta potential converged to neutrality with the increasing polymer dosage, with a more subtle trend for PAM1. The maximum increase was −18.70 and −28.65 at 5000 pm of PAM2 and PAM1, respectively.

The addition of the PAMs, even though they are non-ionic, induced flocculation by bridging particles together through polymer chain adsorption on different particles. H-bonding is believed to be the primary adsorption mechanism between electronegative sites of mineral particles and the nitrogen and carbonyl of the PAM’s amide group [36,55,58,59,60]. The electronegative C=O group of the amide forms an H-bonding with the oxide/hydroxyl groups of the mineral phases present in the iron ore tailings (determined by XRD). In addition, not only the undissolved hydroxyl metals (MOH) are present in the tailings, but also charged groups (MOH_2_^+^ or MO^−^) formed by H^+^ adsorption or desorption, by the hydroxyl groups of the mineral surface. The negative site MO^-^ are more suitable to interact with the primary amine group [58,59]. The inability of PAM to completely neutralize the zeta potential has been attributed to two factors: the steric stabilization effect when H-bonding governs the bridging interactions and the hydrolyzation that can occur during PAM synthesis, which gives them some anionic character [61].

In addition, the small effects of PAM1 on zeta potential increase were due to its lower M_w_, leading to less H-bonding interaction, hydrolyzation, and steric stabilization, if compared to PAM2 (higher molecular weight) [61].

#### 3.3.6. A Comparison with Different Polymer Flocculants

**Figure 18:**
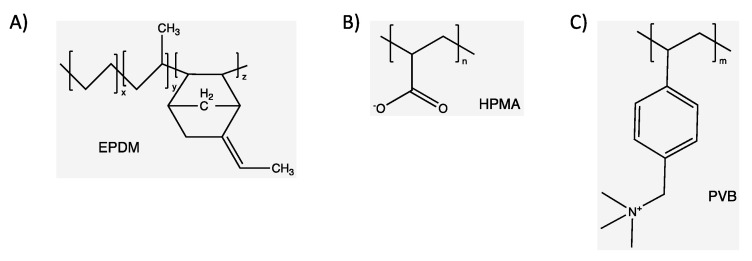
Chemical structure of EPDM (**A**), HPMA (**B**), and PVB (**C**).

We studied the flocculation performance of a 5 wt.% iron ore tailing suspension using two different polymers developed for oil sands tailings. These polymers have been extensively studied to treat oil sand tailings and have already been reported in our previous publications. Poly((vinylbenzyl)trimethylammonium chloride) (PVB) [23,54] is as a linear, cationic, and partially hydrophobic polymer because of its pendant benzene rings. Because of their hydrophobicity, the polymer chains tend to interact strongly with positive charges and negative suspended particles. This creates a sediment with a high solid content. The polymer had a high molecular weight (2.9 × 10^6^ g/mol) and narrow dispersity (*Đ* = 1.19). Partially hydrolyzed poly(methyl acrylate) grafted onto ethylene-propylene-diene copolymer backbones (EPDM-g-HPMA) [22] is a graft copolymer designed to have a certain degree of hydrophobicity provided by the EPDM backbone and negatively-charged functional groups provided by the partially hydrolyzed poly(methyl acrylate) (HPMA). These polymers were synthesized by varying the molecular weight of the EPDM backbone and HPMA grafts. We used a polymer with a molecular weight of 2.1 × 10^5^ and 1.5 × 10^5^ for the backbone and graft, respectively. The chemical structure of the monomers of both polymers is shown in Figure 18.

**Figure 19:**
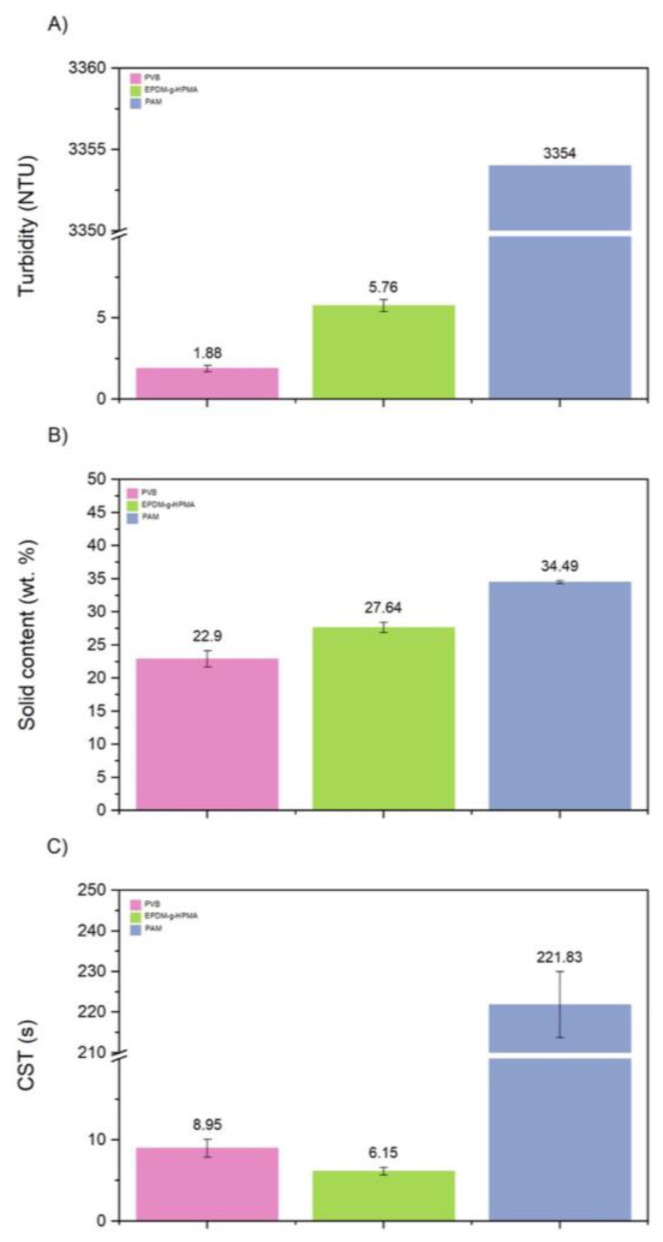
Turbidity, solid content, and CST for a 5 wt.% iron ore tailing treated with PVD (**A**), p(NIPAM- MATMAC-BAAM) (**B**), and EPDM-g-HPMA) (**C**).

**Figure 20:**
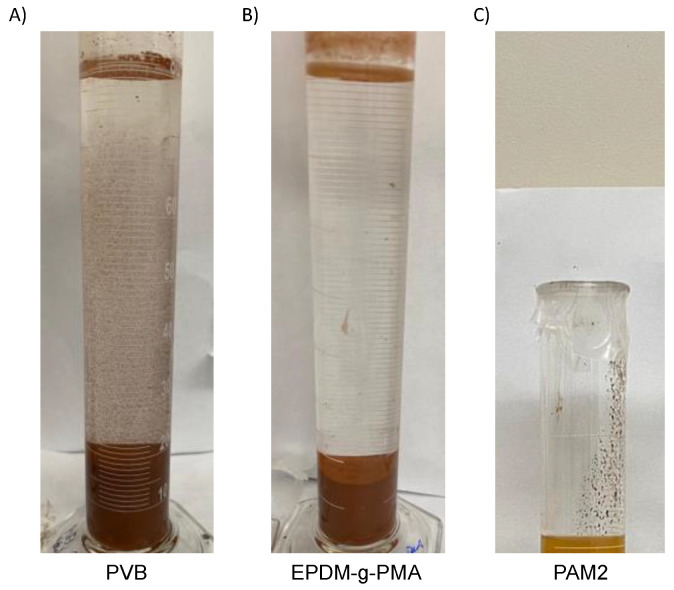
Flocculation performance of 5 wt.% iron ore tailings with different polymers: PVD (**A**), EPDM-g-PMA (**B**), and PAM2 (**C**).

Figure 19 shows the solids content, turbidity, and CST determined for all tests using the same dosage of 1000 ppm. PVB produced supernatants with the lower turbidity (Figure 19A), slightly lower solids content (Figure 19B), and higher CST (Figure 19C). Figure 20 shows that both new polymers clarified the water better than PAM. The positive charges of PVB helped the adsorption of the suspended negative particles, resulting in their destabilization and settling. The higher molecular weight of PVB also boosted the flocculation, resulting in more bridging with fine particles. EPDM-g-HPMA made supernatants with higher turbidity, likely because of its negative charges. Anionic polymers require divalent cations salts to promote bridging between their charged functional groups and the clay’s negatively charged surface.

Figure 19B showed that neither of the new polymers could outperform PAM for solids content. Surprisingly, the partial hydrophobicity of PVB did not improve the densification of the sediments. Similar results were observed by [23] for oil sands tailings flocculation. Regarding EPDM-g-HPMA, the low solid content was likely because of the low molecular weight of the grafts and backbone. Similar results were observed by [22], in which the highest solids content was observed for polymers with the highest molecular weights of the grafts and backbone. Figure 19C also shows that the sediments obtained with EPDM-g-HPMA were easier to dewater (lower CST), possibly because of the hydrophobicity of the backbone.

## 4. Conclusions

We studied the flocculation and dewatering efficiency of two commercially available polyacrylamides, PAM1 and PAM2, using iron ore tailings from the Vargem Grande pond (Nova Lima, Minas Gerais, Brazil). We compared the effectiveness of these polyacrylamides with two novel polymer flocculants: PVB and EPDM-g-HPMA. These polymers were originally designed for flocculating oil sand tailings.

The industrial mineral tailings polyacrylamide (PAM2) has a higher molecular weight than laboratory-scale flocculation polyacrylamide (PAM1), which explains PAM2’s better performance. For both PAM flocculants, the dosage correlated with better separation efficiency. For PAM2, 4000 ppm was found to be the optimal dosage. PAM2 at 2000 ppm produced sediments with the highest solid content (35 wt.%) among all conditions. Because of PAM’s high hydrophilicity, this polymer still retains a large amount of water in the sediments. Sediments with a high water content are less able to resist shear forces, increasing the risk of pond failures.

Despite PAM2’s superior flocculation performance, none of the dosages tested could overcome colloidal stability and clarify the supernatant. While PAM did remove some fine suspended solids, its maximum efficiency was significantly low, making it ineffective at flocculating and dewatering iron ore tailings.

The zeta potential results revealed that although the non-ionic PAMs did not completely flocculate the suspended particles, they did induce some flocculation by bridging the particles together through polymer chain adsorption via H-bonding.

Poly((vinylbenzyl)trimethylammonium chloride) (PVB) and partially hydrolyzed poly (methyl acrylate) grafted onto ethylene-propylene-diene copolymer backbones (EPDM-g-HPMA) were considerably superior to commercially available high molecular weight polyacrylamides. They consistently produced clearer supernatants, despite being developed for completely different tailings. In comparison with PAM, these polymers had the only disadvantage of retaining high amounts of water in the sediment. We may speculate, however, that this constraint could be overcome by increasing the molecular weight of the hydrophobic monomers and varying their proportions.

## Figures and Tables

**Table 1:** GPC results for samples PAM1 and PAM2.

Flocculant	Mw (MDa)	*Đ*
PAM1	5.95	1.11
PAM2	12.49	1.81

**Table 2:** Solid content of the suspended phase from the received tailings, and of the sedimented and suspended phase from the prepared 60 wt.% tailing.

Phase	Solid Content (wt.%)
Suspended phase of the received tailings	11.8 ± 1.4
Sedimented phase of the prepared 60 wt.% tailing	76.8 ± 0.9
Suspended phase of the prepared 60 wt.% tailing	17.2 ± 1.3

**Table 3:** Chemical composition of the sediment and colloidal suspension from 60 wt.% slurry.

Compound	Composition (wt.%)	
	Sediment	Colloidal Suspension
Fe	44.34	47.7
SiO_2_	29.72	3.54
Al_2_O_3_	2.58	7.94
P	0.06	0.345
Mn	0.11	0.7
TiO_2_	0.11	0.48
CaO	<0.10	<0.10
MgO	<0.10	<0.10
Na_2_O	<0.10	1.96
K_2_O	<0.10	<0.10
Cr_2_O_3_	<0.10	<0.10
LF	2.74	15.3

LF = Lost to Fire.

## Data Availability

The data presented in this study are available on request from the corresponding author.
